# Incidence and mortality trends of gastric and colorectal cancers in Croatia, 1988-2008

**DOI:** 10.3325/cmj.2012.53.124

**Published:** 2012-04

**Authors:** Iva Kirac, Mario Šekerija, Iva Šimunović, Lina Zgaga, Danko Velimir Vrdoljak, Dujo Kovačević, Tomislav Kuliš, Ariana Znaor

**Affiliations:** 1 Department of Surgical Oncology, University Hospital for Tumours, University Hospital Center Sestre Milosrdnice, Zagreb, Croatia; 2 Croatian National Institute of Public Health, Zagreb, Croatia; 3 Health Center of Zagreb County, Vrbovec, Croatia; 4 Andrija Štampar School of Public Health, University of Zagreb School of Medicine, Zagreb, Croatia; 5 Center for Population Health Sciences, University of Edinburgh, Edinburgh, UK; 6 Department of surgery, University Hospital Center Sestre Milosrdnice, Zagreb, Croatia; 7 Department of urology, Clinical Hospital Center Zagreb, University of Zagreb School of Medicine, Zagreb, Croatia

## Abstract

**Aim:**

To estimate the incidence and mortality trends of gastric and colorectal cancers in Croatia between 1988 and 2008.

**Methods:**

Incidence data for the period 1988-2008 were obtained from the Croatian National Cancer Registry. The number of deaths from gastric and colorectal cancers was obtained from the World Health Organization mortality database. Joinpoint regression analysis was used to describe changes in trends by sex.

**Results:**

Gastric cancer incidence rates declined steadily during the study period, with estimated annual percent change (EAPC) of -3.2% for men and -2.8% for women. Mortality rates in men decreased, with EAPC of -5.0% from 1988-1995 and -2.5% from 1995-2008. Mortality rates in women decreased, with EAPC of -3.2% throughout the study period. For colorectal cancer in men, joinpoint analysis revealed increasing trends of both incidence (EAPC 2.9%) and mortality (EAPC 2.1%).In women, the increase in incidence was not significant, but mortality in the last 15 years showed a significant increase of 1.1%.

**Conclusion:**

The incidence and mortality trends of gastric cancer in Croatia are similar to other European countries, while the still increasing colorectal cancer mortality calls for more efficient prevention and treatment.

The two most important gastrointestinal cancers in Croatia are gastric and colorectal cancer, which together contribute to about one fifth of Croatian cancer incidence and mortality (1).

## Gastric cancer

Gastric cancer is the fourth most common cancer and the second leading cause of cancer death worldwide (1,2), with high case fatality rate and five-year survival of less than 20% (3). It displays a substantial geographic variability across the continents, with rates in Asia, some parts of Eastern Europe, and Latin America being several times higher than those in western countries (2).

The etiology of gastric cancer has not been entirely elucidated. The most common risk factors reported in the literature are chronic mucosal infection with *Helicobacter pylori* (4), consumption of salt and salt-preserved foods (5), low socio-economic status (6), presence of contaminants in drinking water (7,8), smoking (9), and certain occupational exposures (10,11).

During the second half of the 20th century, incidence and mortality rates of gastric cancer have declined steeply throughout Europe (12,13) and around the world (14), mainly as a result of the remarkable improvement in the living conditions (15-18). This improvement may have contributed to a declining prevalence of *H. Pylori* infection in younger birth cohorts (19) and the use of eradication therapy in older cohorts may have further contributed to the lowering of incidence and mortality rates (20). *H. Pylori* infection plays an important role in the sequence of events that can lead to gastric cancer and is classified as a type I carcinogen according to the International Agency for Research on Cancer criteria (4).

According to the most recent data from the Croatian National Cancer Registry, the gastric cancer crude incidence rate per 100 000 men in 2008 was 29.0 (617 cases), making it the fifth most common cancer; for women the rate was 17.0 per 100 000 (390 cases), making it the eighth most common cancer (21). Among cancer-related causes of death in 2008, gastric cancer ranked fourth both in men, with a crude mortality rate of 24.2 per 100 000 (514 deaths), and in women, with a crude mortality rate of 15.9 per 100 000 (364 deaths) (22).

## Colorectal cancer

Colorectal cancer is a major global public health problem, with approximately 950 000 newly diagnosed cases each year (1). The risk of colorectal cancer increases steeply with age, and in many developed countries colorectal cancer burden is increasing with increasing life expectancy. The incidence is also increasing in many developing countries, as diet and lifestyle become more similar to those in developed countries. Colorectal cancer five-year relative survival in Europe for both sexes combined is 56.2% (23).

Family history of hereditary colorectal cancer is a recognized risk factor, accounting for 15%-20% of cases at the population level (24). Additional 6% excess risk can be attributed to genetic predisposition due to common causal variants in the genome (25-29). Increased colorectal cancer risk is also associated with dietary factors such as high fat and red meat consumption and low vegetable intake, as well as with physical inactivity, excess body weight, and high alcohol consumption (30). On the other hand, it has been inversely associated with the use of exogenous female hormones (31).

Western European countries introduced the secondary cancer prevention in the mid-2000s, most often based on fecal occult blood test followed by colonoscopy (32). Croatia initiated the national colorectal cancer screening program in 2007. Apart from active screening, changes in colorectal cancer trends worldwide could be attributed to standardization and availability of evidence-based surgical, chemo-, and radiotherapy (33,34).

The aim of this study was to analyze gastric and colorectal cancer incidence and mortality trends in Croatia from 1988 to 2008 for both sexes, and to evaluate these findings in comparison to other European countries and in the context of changing environmental and social conditions, with possible implications for the health policies.

## Methods

### Data sources

Incidence data for the period 1988-2008 were obtained from the Croatian National Cancer Registry. The Registry, founded in 1959, covers the whole Croatian population (approximately 4.4 million persons) and relies on mandatory cancer notifications from primary and secondary health care sources and death certificates from the Croatian Bureau of Statistics. The Registry contributed data to the last three volumes of the Cancer Incidence in Five Continents series (35-37). In addition to incidence data, these publications report indices of data quality (proportion of morphologically verified cases, proportion of cases registered from death certificates only, and mortality to incidence ratio). At the Registry, gastric cancer was defined as ICD-9 (38) code 151 from 1988 to 2000 and ICD-10 (39) code C16 from 2001 to 2008. Colorectal cancer was defined as cancer of the hepatic flexure (153.0), transverse colon (153.1), cecum (153.4), appendix (153.5), ascending colon (153.6), splenic flexure (153.7), descending colon (153.2), sigmoid colon (153.3), recto sigmoid junction (154.0), rectum (154.1), and unspecified (153.8 and 153.9) in ICD-9; and the colon (C18.0-C18.9), recto sigmoid junction (C19.0-C19.9), rectum (C20.0-C20.9), and anus (C21) in ICD-10. The number of deaths with gastric and colorectal cancer as the underlying cause were obtained from the World Health Organization mortality database (40). For calculating age-specific rates, we used the population estimates from the Population Division of the Department of Economic and Social Affairs of the United Nations (41).

### Statistical analysis

Age-standardized rates of cancer incidence in Croatia were calculated by the direct standardization method, using the world standard population as a reference (42). To describe incidence and mortality time trends, we carried out joinpoint regression analysis using the software Joinpoint Regression Program, Version 3.5.0. April 2011 (43). The analysis included the logarithmic transformation of the rates, standard error, maximum number of five joinpoints, and minimum of four years between two joinpoints. All other program parameters were set to default values. The aim of the approach is to identify possible joinpoints where a significant change in the trend occurs. The method identifies joinpoints based on regression models with 0-5 joinpoints. The final model selected was the most parsimonious of these, with the estimated annual per cent change (EAPC) based on the trend within each segment (44). To quantify the trend over a fixed period, the average annual per cent change (AAPC) was calculated. The AAPC is computed as a geometric weighted average of the EAPC trend analysis, with the weights equal to the lengths of each segment during the pre-specified fixed interval (45).

In case of non-significant trends (*P* > 0.05), we used the terms “stable” (EAPC between -0.5% to 0.5%), “non statistically significant increase” (EAPC>0.5%), and “non statistically significant decrease” (EAPC<-0.5%). All statistical tests were two sided. Unless otherwise mentioned, the rates were expressed per 100 000.

## Results

### Gastric cancer

During the period 1988-2008, there were 25 183 cases of gastric cancer and 21 313 deaths. The age-standardized incidence rate per 100 000 decreased from 29.5 in the first 5-year period (1988-1992) to 16.9 in the last 5-year period (2004-2008) in men (43%), and from 11.7 to 7.0 in women (40%). The age-standardized mortality rate per 100 000 decreased from 25.2 to 14.8 per 100 000 in men (41% decrease), and from 10.0 to 5.8 in women (42% decrease) (Tables 1 and 2).

**Table 1:** Male gastric cancer incidence and mortality in Croatia in the period 1988-2008. Number of cases, crude rate and age standardized rate (ASR) per 100 000 (using world standard population)

Year	Incidence			Mortality		
	N	crude rate	ASR	N	crude rate	ASR
**1988**	847	39.0	30.9	736	33.9	26.8
**1989**	844	38.8	29.8	752	34.5	26.9
**1990**	872	39.9	31.2	726	33.2	25.9
**1991**	821	37.3	28.6	719	32.7	24.8
**1992**	786	35.5	26.7	621	28.0	21.4
**1993**	704	31.5	23.2	678	30.4	22.3
**1994**	754	33.6	24.1	634	28.2	20.1
**1995**	756	33.6	23.0	635	28.2	19.4
**1996**	720	32.1	21.7	606	27.0	18.4
**1997**	782	35.1	23.1	627	28.1	18.6
**1998**	683	30.9	20.0	610	27.6	17.6
**1999**	831	38.0	24.3	634	29.0	18.4
**2000**	719	33.1	20.8	610	28.1	17.7
**2001**	710	32.9	20.4	573	26.5	16.2
**2002**	711	33.1	20.1	580	27.0	16.3
**2003**	718	33.5	19.9	617	28.8	16.7
**2004**	662	30.9	18.1	610	28.5	16.4
**2005**	667	31.2	17.8	556	26.0	14.9
**2006**	604	28.3	16.1	539	25.3	14.1
**2007**	628	29.5	16.4	578	27.1	15.3
**2008**	617	29.0	16.0	514	24.2	13.1

**Table 2:** Female gastric cancer incidence and mortality in Croatia in the period 1988-2008. Number of cases, crude rate, and age standardized rate (ASR) per 100 000 (using world standard population)

Year	Incidence			Mortality		
	N	crude rate	ASR	N	crude rate	ASR
**1988**	541	23.4	12.1	482	20.8	10.8
**1989**	527	22.7	12.0	458	19.7	10.5
**1990**	556	23.8	12.9	469	20.1	10.8
**1991**	503	21.4	11.2	432	18.4	9.6
**1992**	460	19.4	10.2	372	15.7	8.1
**1993**	436	18.2	9.1	398	16.6	8.3
**1994**	447	18.5	9.5	388	16.1	8.1
**1995**	504	20.8	10.1	394	16.3	8.0
**1996**	494	20.5	9.9	408	16.9	8.0
**1997**	494	20.6	10.3	393	16.4	7.9
**1998**	432	18.2	8.5	389	16.4	7.6
**1999**	466	19.8	9.2	349	14.8	6.5
**2000**	516	22.1	10.3	359	15.4	6.7
**2001**	474	20.4	9.0	374	16.1	6.9
**2002**	474	20.5	9.1	395	17.1	7.4
**2003**	435	18.8	7.7	378	16.4	6.7
**2004**	430	18.6	7.8	357	15.5	6.2
**2005**	397	17.2	7.0	350	15.2	6.1
**2006**	374	16.3	6.5	317	13.8	5.4
**2007**	397	17.3	6.8	332	14.5	5.3
**2008**	390	17.0	6.9	364	15.9	6.2

**Figure 1:**
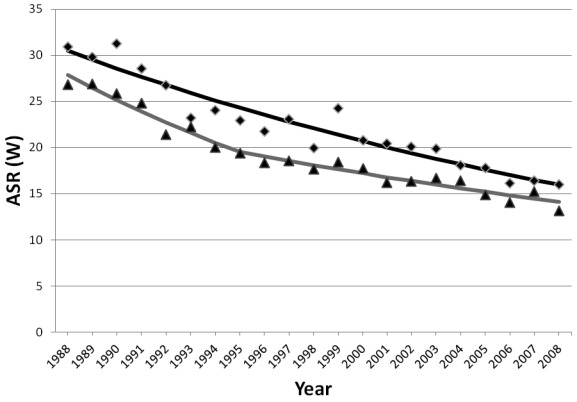
Joinpoint analyses of incidence (rhombs) and mortality (triangles) for gastric cancer in Croatia in the period 1988-2008 for men. ASR (**W**) – age standardized rate per 100 000 (world standard population).

**Figure 2:**
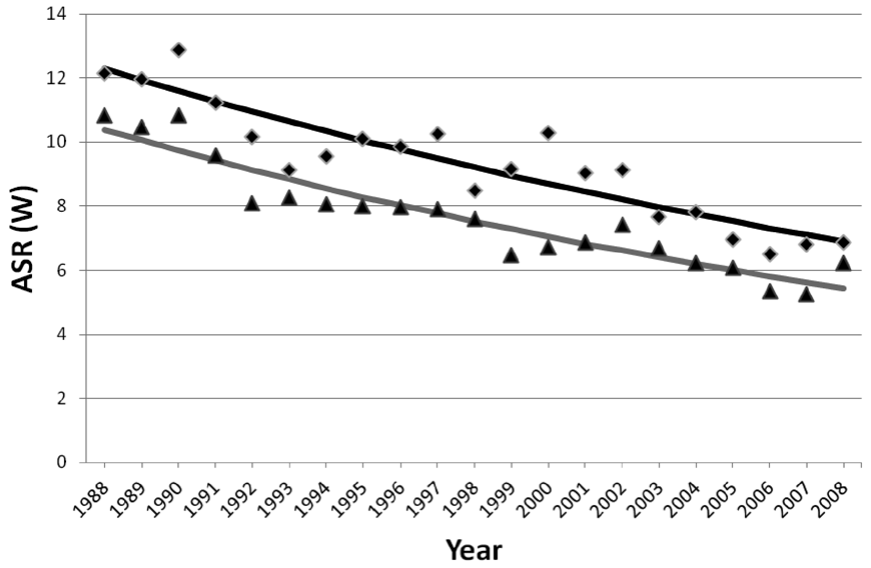
Joinpoint analyses of incidence (rhombs) and mortality (triangles) for gastric cancer in Croatia in the period 1988-2008 for women. ASR (**W**) – age standardized rate per 100 000 (world standard population).

In both sexes, gastric cancer incidence and mortality rates declined steadily (Figure 1 and 2). In men, incidence rates decreased over the entire period (EAPC -3.2%; 95% confidence interval [CI], -3.6 to -2.7), with the best fitting model having no joinpoints, while mortality rates decreased sharply between 1988 and 1995 (EAPC -5.0%; 95% CI, -6.5 to -3.4) and then less sharply between 1995 and 2008 (EAPC -2.5%; 95% CI, -3.1 to -1.8). In women, incidence rates decreased (EAPC -2.8%; 95% CI, -3.5 to -2.2), also with no joinpoints in the final model, and the mortality rates decreased over the entire period (EAPC -3.2%; 95% CI, -3.7 to -2.7) (Table 3).

**Table 3:** Trends in incidence and mortality of gastric cancer in Croatia according to joinpoint analyses, 1988-2008, by sex, with the estimated annual percent change (EAPC) and 95% confidence interval (CI)

	Joinpoint analyses							
	trend 1				trend 2			
	years	EAPC		95% CI	years	EAPC		95% CI
Men:								
incidence	1988-2008		-3.2	-3.6 to -2.7				
mortality	1988-1995		-5.0	-6.5 to -3.4	1995-2008		-2.5	-3.1 to -1.8
Women:								
incidence	1988-2008		-2.8	-3.5 to -2.2				
mortality	1988-2008		-3.2	-3.7 to -2.7				

### Colorectal cancer

**Figure 3:**
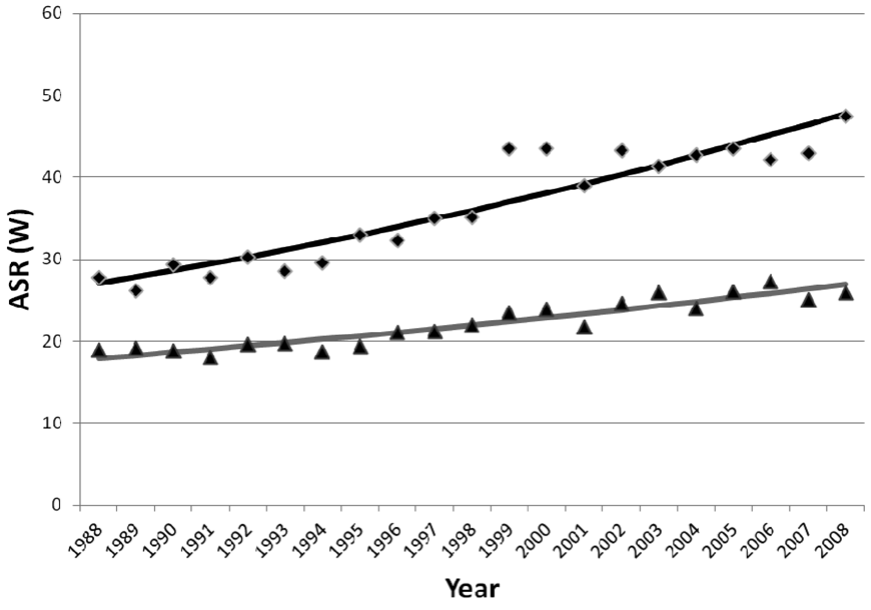
Joinpoint analyses of incidence (rhombs) and mortality (triangles) for colorectal cancer in Croatia in the period 1988-2008 for men. ASR (**W**) – age standardized rate per 100 000 (world standard population).

From 1988 to 2008, there were 25 964 cases of colorectal cancer in men (Table 4). The number of male colorectal cancer cases increased more than 2-fold, from 767 in 1988 to 1818 in 2008, while ASR increased from 27.8 to 47.5. In that period, 15 983 male colorectal cancer patients died. The number of colorectal cancer deaths doubled, from 509 in 1988 to 1052 in 2008, while ASR increased from 19.0 to 26.0/100 000. Joinpoint analysis in men did not identify any joinpoints (Table 5 and Figure 3). The incidence trend significantly increased (EAPC 2.9%; 95% CI, 2.3 to 3.9%), as well as mortality trend (EAPC 2.1%; 95% CI, 1.7 to 2.4%).

**Table 4:** Male colorectal cancer incidence and mortality in Croatia in the period 1988-2008. Number of cases, crude rate, and age standardized rate (ASR) per 100 000

Year	Incidence			Mortality		
	N	crude rate	ASR	N	crude rate	ASR
**1988**	767	35.3	27.8	509	23.4	19.0
**1989**	729	33.5	26.2	535	24.6	19.2
**1990**	837	38.3	29.3	523	23.9	18.8
**1991**	780	35.5	27.7	522	23.7	18.0
**1992**	886	40.0	30.2	582	26.3	19.6
**1993**	876	39.2	28.5	601	26.9	19.7
**1994**	947	42.2	29.6	601	26.8	18.7
**1995**	1096	48.7	33.0	631	28.0	19.4
**1996**	1076	48.0	32.3	702	31.3	21.1
**1997**	1172	52.6	35.0	718	32.2	21.2
**1998**	1198	54.3	35.1	758	34.3	22.0
**1999**	1498	68.5	43.5	801	36.6	23.5
**2000**	1503	69.3	43.5	841	38.8	23.9
**2001**	1369	63.4	39.0	770	35.7	21.8
**2002**	1544	71.8	43.3	885	41.2	24.6
**2003**	1482	69.1	41.3	956	44.6	26.0
**2004**	1556	72.6	42.7	898	41.9	24.1
**2005**	1609	75.2	43.5	998	46.7	26.0
**2006**	1595	74.7	42.1	1028	48.2	27.3
**2007**	1626	76.3	42.9	982	46.1	25.1
**2008**	1818	85.5	47.5	1052	49.5	26.0

**Table 5:** Trends in incidence and mortality of colorectal cancer in Croatia according to joinpoint analyses, 1988-2008, by sex, with the estimated annual percent change (EAPC) and 95% confidence interval (CI)

	Joinpoint analyses								
	Trend 1			Trend 2			Trend 3		
	years	EAPC	95% CI	years	EAPC	95% CI	years	EAPC	95% CI
Men:									
incidence	1988-2008	2.9	2.3 to 3.5						
mortality	1988-2008	2.1	1.7 to 2.4						
Women:									
incidence	1988-1996	-0.2	-1.9 to 1.6	1996-1999	10.6	-3.7 to 27.1	1999-2008	-0.5	-1.7 to 0.7
mortality	1988-1994	-1.9	-4.1 to 0.3	1994-2008	1.1	0.5 to 1.7			

**Figure 4:**
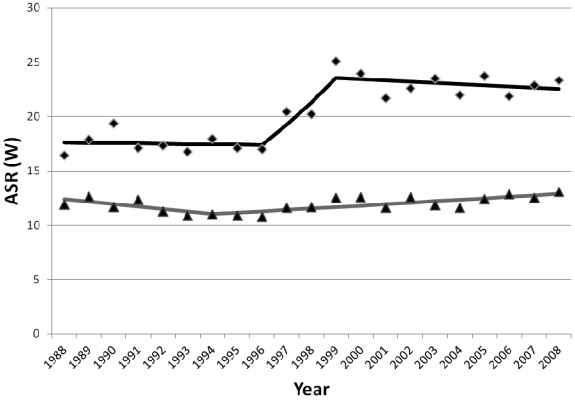
Joinpoint analyses of incidence (rhombs) and mortality (triangles) for colorectal cancer in Croatia in the period 1988-2008 for women. ASR (**W**) – age standardized rate per 100 000 (world standard population).

There were 20 752 cases of female colorectal cancer (Table 6). From 1988 to 2008, the number of cases increased almost 2-fold, from 685 to 1254. The ASR increased from 16.4/100 000 to 23.4/100 000. In the same period, 12 999 female colorectal cancer patients died. The number of colorectal cancer deaths increased from 508 in 1988 to 803 in 2008, while ASR increased from 11.9/100 000 to 13.1/100 000. Joinpoint analysis of female colorectal cancer incidence trends (Table 5 and Figure 4) identified two joinpoints, in 1996 and 1999, resulting in three separate trends: from 1988 to 1996 the trend was stable (EAPC -0.2%; 95% CI, -1.9 to 1.6%), from 1996 to 1999 the trend showed a non-significant increase (EAPC 10.6%; 95% CI, -3.7 to 27.1%), and from 1999 to 2008 the trend was again stable (EAPC -0.5; 95% CI, -1.7 to 0.7%). AAPC for the period from 1996 (when the first increase in incidence was noted) to 2008 showed a non-significant increase (AAPC 2.2%; 95% CI, -1.1 to 5.6%). Joinpoint analysis of female colorectal cancer mortality identified one joinpoint in 1994, resulting in two separate trends: the trend from 1988 to 1994 showed non-significant decrease (EAPC -1.9%; 95% CI, -4.1 to 0.3%), while from 1994 to 2008 it showed a significant increase (EAPC 1.1%; 95% CI, 0.5 to 1.7%). 

**Table 6:** Female colorectal cancer incidence and mortality in Croatia in the period 1988-2008. Number of cases, crude rate, and age standardized rate (ASR) per 100 000 using world standard population

Year	Incidence			Mortality		
	N	crude rate /100 000	ASR /100 000	N	crude rate /100 000	ASR /100 000
**1988**	685	29.6	16.4	508	21.9	11.91
**1989**	770	33.2	17.9	556	24.0	12.66
**1990**	811	34.8	19.4	525	22.5	11.68
**1991**	734	31.3	17.1	545	23.2	12.34
**1992**	766	32.3	17.4	518	21.8	11.27
**1993**	755	31.5	16.8	518	21.6	10.90
**1994**	827	34.3	17.9	530	22.0	10.97
**1995**	823	34.0	17.1	540	22.3	10.88
**1996**	824	34.2	17.0	553	22.9	10.79
**1997**	963	40.2	20.5	570	23.8	11.60
**1998**	986	41.5	20.2	604	25.4	11.67
**1999**	1186	50.4	25.1	639	27.2	12.50
**2000**	1197	51.3	24.0	668	28.6	12.59
**2001**	1072	46.2	21.7	640	27.6	11.62
**2002**	1116	48.2	22.6	673	29.1	12.59
**2003**	1213	52.5	23.5	666	28.8	11.84
**2004**	1143	49.5	22.0	666	28.9	11.59
**2005**	1237	53.7	23.7	749	32.5	12.38
**2006**	1175	51.1	21.9	772	33.6	12.84
**2007**	1215	52.9	22.9	756	32.9	12.51
**2008**	1254	54.7	23.4	803	35.0	13.07

## Discussion

This is the first study to systematically analyze the time trends of gastric and colorectal cancer incidence and mortality in Croatia. We chose to use joinpoint regression modeling as the optimal method to detect and depict sharp changes in trends, reflecting changes in cancer prevention and care policies.

### Gastric cancer

Our analysis revealed a persistent and steady decrease in gastric cancer incidence and mortality rates in Croatia between 1988 and 2008, with rates at the end of the study period being ~ 40% lower than at the beginning. The decrease was observed in both men and women, and the trends did not level off in the recent years.

Croatia is in the upper half of the list of European countries according to age-standardized gastric cancer incidence and mortality rates for both men and women. According to the GLOBOCAN database, the highest incidence and mortality rates in Europe are observed in Belarus, Albania, and Russian Federation (incidence rates between 25-34/100 000 in men and 12-18/100 000 in women; and mortality rates between 21-30/100 000 in men and 10-15/100 000 in women), with much higher rates in Central and Eastern European countries than in more developed Western or Northern European countries (1).

In the EU from 1980 to 1999, a steady and persistent decrease in gastric cancer mortality rates was observed (from 18.6 to 9.8/100 000 in men and from 8.9 to 4.6/100 000 in women, ~ 50% for each sex) (13,46). Decreases were also reported between 2000 and 2005 (47). The reasons for the decrease are complex, and not completely understood. They include more varied and better nutrition, better food preservation (refrigeration), decreased prevalence of *H. pylori* infection due to improved living conditions and changes in lifestyle (48-50), and, at least to some extent, decreased smoking prevalence in men (51). The lowering of incidence rates leads to lowering of mortality rates, with a possible contribution of improved medical treatment (52).

During the second half of the twentieth century, Croatian society experienced both industrial and nutritional changes. The availability of various food products, such as fruits and products of animal origin, was facilitated by the introduction of refrigerators (53). Progressive globalization after the war in Croatia (1991-1995) led to a smaller need for homemade food production, which was heavily salted, marinated, and low in antioxidants (54).

The prevalence of *H. Pylori* infection in Croatia in 1998 in a random sample of individuals aged 20-70 was 60.4%, with higher rates in the southern parts of the country (55). This is considered to be an average value, since the prevalence ranges from 30% in the western countries to 60%-88% in Asian countries (56). Unfortunately, more recent data for Croatia are not available.

The prevalence of smoking in 1995 was 34% among men and 27% among women in 18-65 age group (57), while the prevalence in 2003 was 34% among men and 22% among women in 18+ age group (58). The relatively stable proportion of smokers in Croatia may indicate that the lowering of gastric cancer rates was not influenced by changes in smoking habits.

The use of joinpoint regression model allows a fresh approach to interpretation of trends in cancer mortality over a longer time period. This study, as well as other studies of gastric cancer trends (13,14), shows a decline in mortality, with no signs of leveling off in the recent years. Thus, it is expected that the decrease in incidence and mortality rates will continue (59), especially because such decreases are also observed in the countries that already have relatively low gastric cancer incidence and mortality rates, such as Iceland, Cyprus, and Sweden (incidence rates between 4.7-6.6/100 000 in men and between 2.5 and 3.1/100 000 in women; and mortality rates between 4.1-4.2/100 000 in men and between 1.8-2.4/100 000 in women) (1).

### Colorectal cancer

The incidence trend of colorectal cancer showed a significant increase in men (EAPC 2.9%). In women, there was no significant change in trend, though there was a constant non-significant increase. In 2008, with 1818 new cases colorectal cancer was the third most common cancer in Croatian male population and with 1052 deaths, the second cause of cancer mortality (21). In women, with 1254 new cases it was the second most common cancer, and with 803 deaths the second cause of cancer death. Compared to GLOBOCAN 2008 estimates for Europe, Croatia ranked ninth according to incidence of colorectal cancer (ASR 44.4/100 000) in men and fifteenth (ASR 24.3/100 000) in women, but fourth according to mortality in both men (ASR 25.4/100 000) and women (ASR 13.1/100 000) (1).

Incidence trends vary across Europe, with persistent increases or no change in Eastern European countries and persistent slight decreases in Western Europe (17). The increase in the incidence in Eastern Europe (eg, Slovakia or Czech Republic, as well as Croatia) could be attributed to lifestyle changes, presumably introduced by westernization, resulting in obesity and physical inactivity (60). In fact, in 2003 over 1.3 million people in Croatia were either obese or overweight (61). The observed variations of the incidence rates of colorectal cancer reflect the distributions of risk factors across European countries, as well as disparities in the effective cancer prevention and detection (62). However, such results should be interpreted with caution since all registries from which the data have been obtained do not comply with quality requirements, and a substantial number of Eastern European countries eg, Greece, Bosnia and Herzegovina, FYR Macedonia, and Romania, do not have established national cancer registration (63).

In the European Union, colorectal cancer mortality has been declining since the early 1980s by around 1% per year in women, and since the early 1990s by 0.6% per year in men (64). Decreases were found in Austria, Finland, Ireland, Netherlands, Norway, Sweden, Switzerland, United Kingdom, France, and Italy, and even earlier in Belgium, Germany, and Denmark (65). On the other hand, increases were observed in Bulgaria, Poland, Romania, Greece, Portugal, and Spain. In the Czech Republic, Slovakia, and Hungary, the rates were exceedingly high, but remained constant in the recent years (65).

Incidence and the mortality rates may also be affected by screening and early detection. All but three countries (Greece, Russia, and Turkey) (66-68) participate in some form of colorectal cancer screening. However, only five European countries have an organized population-based screening program (Croatia, Finland, France, Italy, the UK) (69-72) and only five have screening guidelines – Finland, France, Germany (73), Italy, and the UK. There is still room for improvement since the participation rate in the voluntary screening is 15% and up to 60% in the organized screening programs (74). In the Croatian national screening, the participation rate was 19.9% (69).

Several randomized trials and one Cochrane review provided strong evidence that fecal occult blood test followed by colonoscopy, if offered every 2 years, reduced mortality rates associated with colorectal cancer by 16% (75,76). In the United States, where screening was introduced in the 1970s, long-term screening programs have played a relevant role in reducing colorectal cancer incidence and mortality over the last two decades. One of the explanations for better survival rates in the United States is an increased detection and removal of colorectal polyps, which often precede colorectal cancer development. Screening programs have been implemented in Europe, including Croatia, only recently, so their impact on the incidence and mortality may become more visible in the future (76). Nevertheless, increased patient awareness of signs and symptoms, recognition in the primary care, and access to diagnostic modalities may have already played a role.

In the past ten years, countries across the EU have initiated national policies for improvement in diagnostics, referral, and assurance of quality of care in specialized centers with attending multidisciplinary cancer teams. The main goals were to improve survival and quality of life and to reduce regional differences in treatment options. Following the publication of the Campbell Report, the UK has implemented specialization of cancer services, which proved to be closely related to a reduction in mortality. Another important step is the shortening of the period between the symptom appearance and the referral to diagnostics (33).

Another important factor that explains the observed patterns of survival and mortality, alongside screening, is the progress in therapy (77). The general improvement in surgical techniques for localized and metastatic tumors substantially prolonged survival (78,79). Only the adoption of surgical training (subspecialization) and pathological evaluation of specimens (quality control) in rectal cancer management resulted in an overall improvement in survival (34,80,81). The wider adoption of new treatment protocols and adjuvant therapies, including progressive increase in sequential adjuvant chemotherapy for advanced non-localized tumors, and preoperative chemo- and radiotherapy for rectal cancers (82), may have favorably influenced colorectal cancer mortality (47,77,83,84). Moreover, in the past fifteen years five new drugs have been introduced (85,86).

We believe that the discrepancy between the incidence (9th in male and 15th in female colorectal cancer) and mortality (4th for both sexes) rank of Croatia in Europe strongly indicates the need for improvement of prevention and treatment.

There are still no standardized protocols for treatment and referral, or requirements for the availability of multidisciplinary teams for colorectal cancer treatment in most Croatian hospitals, with the exception of university hospitals. Due to the high incidence and increasing mortality, Croatian Society of Coloproctology and Croatian Society of Oncology have launched an incentive for quality control and referral improvements for colorectal cancer patients, recognizing the current lack of availability of all treatment modalities at different hospitals (87). Hopefully, establishing accrediting centers for colorectal cancer treatment with trained staff would ensure that every patient receives optimal surgical, neo adjuvant or adjuvant treatment, systemic and local (radiotherapy), and regular follow-up.
